# Observations on the Lepra Arabum, or Elephantiasis of the Greeks, as It Appears in India

**Published:** 1826-04

**Authors:** 


*Observations on the Lepra Arabum, or Elephantiasis of the Greeks, as it appears in India.* By WHITELAW AINSLIE, M.D. M.R.A.S. From the first volume of the Transactions of the Royal Asiatic Society, 1826.

THIS paper of Dr. Ainslie's, as might be expected from the great learning of the Author, contains many curious researches respecting the name, history, nature, and treatment of this dreadful and loathsome complaint. We regret that our limits will not permit us to indulge in extracts from the literary disquisitions and erudite researches of this truly talented and learned physician; but compel us to keep as close as possible to the subjects of nature and treatment. It would appear that this disease varied in respect to prevalence, at different æras, even in the same countries. Thus Galen avers, that it was very common in Egypt, in his time, though Savary says he never saw an instance of it while he travelled through that country. In India it is very prevalent, and the most piteous wretches are often seen covered with scurf, or deprived of their fingers and toes. Dr. Ainslie considers the definition of Sauvages as the best. "Facies deformis tuberibus callosis, ozœna, raucedo, cutis Elephantina, crassa, unctuosa, in extremis artubus anæthesia." Cullen omits an essential symptom, ozœna, which is never absent in the advanced stages. Dr. A. is very doubtful that it is at all contagious, in which he is confirmed by the testimony of the best Tamool doctors, who deny any infectious quality in the disease. Of three Europeans who died of elephantiasis, under Dr. A. none of the wives or servants became affected with the complaint. There is every reason to believe, however, that this species of Leprosy is hereditary—at least, it is very common for children, born after the malady has commenced in the parents, to be attacked by it. The Hindu doctors have no doubt of its transmission in this way. Some authors, as Hillary, Sonneni, and Bancroft, have noticed the salacity of lepers; while Adams, and some others have observed a wasting of the testicles. In two instances which we lately saw in this country—one at Brighton, there was this wasting of the testicles. Such a circumstance, however, is not incompatible with their having families—at least during the early stages of the disease, before the general debility becomes excessive. In India, Elephantiasis is by no means of rare occurrence, sparing neither caste nor sect, though much more commonly found among the poor than the rich, for obvious causes. It seldom shews itself before puberty—but when it does, it wonderfully represses the growth of the body. They soon become meagre, shrivelled, and miserable, with shrill and nasal voices. With coming years they evince little sexual desire, the beard either not appearing, or being of a very delicate texture. The mind, of course, is curtailed of its fair proportions, in correspondence with the body. The malady generally begins its depredations about the age of 23 or 24 years—seldom later than forty, and the following are the symptoms, according to Dr. Ainslie's observations, which mark the approach, progress, and termination of this frightful disease.

"The unhappy person fated to perish by this slow but relentless affliction, first perceives an unusual dryness and slight roughness of skin in his hands, feet, arms, and legs, which, even after violent exercise, do not transmit the perspiration readily; he begins to fall off a little in his appetite, and to be much troubled with flatulence and other signs of indigestion, but he is as yet not ill enough to be alarmed, and pursues his customary occupation; his sleep, soon after this, in place of being refreshing to him as it used to be, is disturbed by wild dreams, and he frequently during the night starts up in a fright, with a palpitating heart and sense of suffocation. About six weeks or two months from the time of his first being taken ill, his colour begins to change; if he was rather a fair man, he grows at least two shades darker, and his features lose much of their natural aspect, becoming somehow tumid and less agreeable than formerly. The dryness and roughness of skin increase, and about the end of the third month he complains of a strange numbness in his hands and feet, which he can allow to be pinched without feeling pain; his pulse, which was most likely always feeble, will, if felt, be found to be extremely languid, small, nay, at times, scarcely to be perceived. The aridity and uneveness of skin now extend further, reaching as high as the middle of the arm and leg; indeed, the cuticle over the whole body seems rigid, harsh, and to have entirely lost that smooth and healthy look which it had before the lepra made its primary attack. About this period many dark coloured spots and purple tubercles usually appear on the ancles and wrists, and partially on the legs and arms; they are in shape not unlike segments of ripe currants, but flatter at top, and of a singular shining and oily aspect; they are not attended however with any pain, neither are they particularly itchy, which in truth they could not well be, when we consider that they are subsequent to the want of feeling which I have above described. Some of the tubercles occasionally disappear suddenly, and return again, without evident cause; others generate a small quantity of ichorous matter, which drying, occasions a trifling scurfy desquamation. At this stage of the malady I have met with one or two cases in which glandular swellings at the upper and inner part of the thigh made their appearance, similar to those mentioned by Dr. Adams; but, as far as I can learn, this is by no means so constant a symptom of the disease in India as it seems to be in Madeira. The leprosy advancing, the tubercles increase in size and number, and seizing on the face, render the infected person a most unsightly object. It must here be remarked, that up to this period the breast, abdomen, and back either remain tolerably smooth, or the tubercles are comparatively much fewer upon them; they are moreover smaller in size, nor ever on those parts do they occasion much white desquamation, the natural consequence of their greater vitality. About the end of the first year every symptom is much aggravated: the dryness and rigidity of skin becomes universal, is distressing in the greatest degree; the numbness has extended to above the knee, and is so great, that the poor sufferer may, through inadvertency, burn his hands or feet to the bone without perceiving it: the surface of the whole frame assumes a bright yet unctuous appearance; when narrowly examined, it looks wrinkled longitudinally, and not unfrequently feels, in those parts where feeling remains, as if stung with nettles, rising up into wide spreading irregular bumps, which come and go. The skin about the wrists and ancles, where the tubercles have scaled off, has a scurfy appearance, and here and there a raw excoriation may be perceived, the consequence, perhaps, rather of chaffing than ulceration. The countenance alters still more; the cheeks grow bloated and puffy, and are studded, if I may so say, with irregular dark protuberances; the muscles of the forehead enlarged, seem as if pushed downwards; the eyebrows, thickened and swollen, hang over the eyes, which being in every instance inflamed and rheumy, and having been made to look rounder by the pressure from the neighbouring parts, resemble those of some wild animal; the lobes of the ears are rough, knotty, and mis-shapen; the tongue is foul, and is in some cases blistered with tubercles, which bleed; the breath is fœtid; the voice sounds unpleasant; the urine is plentiful, and generally turbid, having a most unnatural odour; the bowels irregular; the hairs of the head gradually fall off; the parts of generation shrink; the nails break and waste away; the fingers and toes seem as it were withered, the former bending inwards as if crampt, and the heels and soles of the feet are disfigured by deep fissures. The disease gradually going on, and the humours of the body becoming, from the impeded transpiration and general stagnation, daily more corrupt; the voice, which was but six months before only unpleasant, owing perhaps to tubercles on the uvula and palate, has now a most discordant, nasal, and unnatural sound; the *alæ nasi* are swelled and scabrous, and the bones themselves of that organ are in certain cases flattened, and twisted in some degree to one side, giving to the countenance a distorted look. A most offensive ichor now distils from the nose; neither rest nor food tend to refreshen or invigorate, and all carnal appetite, in place of being increased, as some authors imagined, entirely dies away.

" In this condition, with many of the grand functions which support life deranged, it may easily be imagined that existence must be a state of misery; and the conviction that there is no hope whatever of recovery, makes the wretched leper still more an object of pity.

" In the advanced state to which I have brought, in description, the Lepra Arabum, as it appears in India, the malady will sometimes continue for several years, apparently having come to an ultimate stand; but, alas! with declining years is sure to come progressive misery: every symptom is finally rendered worse; the already ugly become loathsome; on the most trifling motion the respiration is hurried, and the dyspepsia is most tormenting, owing in all probability to the perspiration being obstructed over so great a part of the surface of the body, and the certain accumulation of morbific humours: when any exertion is used sufficient to excite diaphoresis, the only parts that perspire are the neck and a little round the waist; the face, legs, arms, and thighs are thereby merely rendered clammy, and the tubercles on them turgid. At this time a feverish attack comes on regularly every evening, which may be discovered by the increased heat of the axilla, and the eyes assume that dim but brassy appearance, so properly noticed by Aretæus; pulsation is no longer felt any where, but by pressure over the heart itself; the whole frame is emaciated, the face is frightful to behold, the voice sounds hollow as if from the tomb: the hands and feet now, from long want of due nourishment, begin to give way; partially blistered-looking ulcerations taking place over their joints, they gradually drop off, and so add helplessness to misery and long-protracted calamity. Soon after this stage, comes the last closing scene; worn out by lingering and hopeless wretchedness, dead almost to every feeling of body as well as mind, the poor leper hastens to his grave: yet, cadaverous as he is, he is not deserted in his expiring moments, but finds a humane and charitable support from the more prosperous of his race. If a Pariah, he is taken care of by those of the same rank till death comes to his relief: if a Hindu or Muhammedan, he is cherished by the individual benevolence of his sect or caste; and having been conveyed to the vicinity of some pagoda or mosque, breathes out his dying prayer on what he conceives to be sacred ground!" 12.

The above most excellent graphic delineation of elephantiasis appertains, of course, to the disease as it appears unchecked by medical aid. It is, however, modified in individuals by peculiarity of constitution or other idiosyncrasy. In poor and badly fed people, whose circulation is languid, and whose stamina are weak, the lepra will soon reach to its greatest height —whilst among the upper classes, its progress will be much retarded. Our author cannot agree with Mr. Robinson (Med. Chir. Trans. vol. x.) in making two varieties of this disease—the one characterised by want of feeling in the extremities—the other by tubercles. Dr. A. has never met with a case of the genuine disease which was not distinguished by both these peculiarities.

Dr. A. has already expressed a doubt as to any contagious property in the disease—but assigned reasons for believing it to be hereditary. But there is another question—can lepra occur independently of any constitutional predisposition? Dr. A. is inclined to think it may occur, under a particular combination of causes, in most regions of the Torrid Zone. It is a curious and singular fact that, in every instance of lepra which Dr. A. has seen in the European—that European was either a German, a Dane, or a Swede—"but never an Englishman." This is comfort for John Bull. From the investigations of Mr. Stewart, stationed at Tranquebar, where lepra is very common, the following results were obtained.

"1st. That women are less liable to suffer from Elephantiasis than men.

"2d. That the disease is most certainly hereditary.

"3d. That its being in any degree contagious is extremely problematical.

"4th. That every leper, suffering from an advanced stage of the malady, doubts whether he is capable of propagating his species.

"5th. That a fish diet is found to render every symptom worse.

"6th and lastly. That poor living, want of cleanliness, mendicant misery, and exposure to cold and damp, are but the too constant attendants of this dreadful affliction." 15.

We deem it useless to notice the etiological speculations of various authors—since none of them appear to rest on any solid foundation.

*Treatment.* In this, as in all other hereditary complaints, much good may be done by avoiding what has been termed the exciting causes. As to a *cure*, when the disease is actually formed, there is little chance of that. The modern Arabian physicians trust chiefly to mercury. Dr. Hillary avoided mercury, and prescribed sarsaparilla. Dr. Towne thought that antimonial medicines afforded the greatest relief, and that mercury aggravated the complaint. Dr. Ainslie always endeavoured, in the first place, to improve the general health of the patient by nourishing diet, exercise, and cleanliness. Then he appears to think that a cautious trial may be made with the oxymurias hydrargyri, in conjunction with warm bathing—and, when we have done our utmost by these means, we are to endeavour to support the frame by generous wine or other cordial. The mineral acids are, he thinks, of unquestionable service in this dreadful malady. So is the vinum antimonii compositum of the Pharmacopœia Chirurgica. The Hindu practitioners, for ages past, have considered the white oxide of arsenic as a powerful remedy in lepra arabum. One author was disappointed in the trials he made with this medicine.

"But of all the alterative and deobstruent remedies employed by the native practitioners of India in this complaint, none is of equal repute with the concrete milky juice of the plant called by the Tamools *Yercam* (Asclepias Gigantea); it exudes from the leaves and tender shoots on being pricked, and has at first somewhat the appearance of cream; but on drying becomes a little darker coloured, and has a rather nauseous and acrid taste; the dose is about a quarter of a gold pagoda weight, given twice daily, together with a little sulphur, and continued for some weeks." 21.

The pathology of this disease is involved in much obscurity. With the following short extract, we must conclude our notice of Dr. Ainslie's interesting Essay.

"The appearances of the body on dissection do not throw much light on the peculiar nature of the malady, further than that I have observed in such cases the heart to be usually small, and the arterial system altogether shrunk and collapsed: the liver I have in one or two instances found indurated, and the gall bladder for the most part distended with viscid and very dark coloured bile; the contents of the abdomen had, generally speaking, an unusually pale and wasted appearance: the bones, when laid bare, were dry and brittle; the testicles, in one or two instances, were almost entirely obliterated; and, on opening the head, it has appeared to me that there was a more than ordinary determination of the blood to the membranes of the brain." 23.

